# TIMELESS Promotes Tumor Progression by Enhancing Macrophages Recruitment in Ovarian Cancer

**DOI:** 10.3389/fonc.2021.732058

**Published:** 2021-08-19

**Authors:** Xin Xing, Fei Gu, Lanyu Hua, Xiaoxiao Cui, Dongxue Li, Zhiyong Wu, Rong Zhang

**Affiliations:** ^1^Department of Obstetrics and Gynecology, Fengxian Hospital Affiliated to the Southern Medical University, Shanghai, China; ^2^The Third School of Clinical Medicine, Southern Medical University, Guangzhou, China; ^3^Shanghai Cancer Institute, Shanghai, China; ^4^Gynecology Department, Shanghai Obstetrics and Gynecology Hospital of Fudan University, Shanghai, China

**Keywords:** ovarian cancer, macrophages, chemokines, bioinformatic analysis, TIM

## Abstract

**Objective:**

Ovarian cancer (OV) is the most fatal and frequent type of gynecological malignancy worldwide. TIMELESS (TIM), as a circadian clock gene, has been found to be highly expressed and predictive of poor prognosis in various cancers. However, the function of TIM in OV is not known. This study was designed to investigate the biological functions and underlying mechanisms of TIM during OV progression.

**Methods:**

Cell viability assay, cell migration assay, immunohistochemistry staining, qPCR analyses, and tumor xenograft model were used to identify the functions of TIM in OV. Bioinformatics analyses, including GEPIA, cBioPortal, GeneMANIA, and TIMER, were used to analyze the gene expression, genetic alteration, and immune cell infiltration of TIM in OV.

**Results:**

TIM is highly expressed in OV patients. TIM knockdown inhibited OV cell proliferation, migration, and invasion both *in vitro* and *in vivo*. Genetic alteration of TIM was identified in patients with OV. TIM co-expression network indicates that TIM had a wide effect on the immune cell infiltration and activation in OV. Further analysis and experimental verification revealed that TIM was positively correlated with macrophages infiltration in OV.

**Conclusions:**

Our study unveiled a novel function of highly expressed TIM associated with immune cell especially macrophages infiltration in OV. TIM may serve as a potential prognostic biomarker and immunotherapy target for OV patients.

## Introduction

Ovarian cancer (OV) is the second leading cause of gynecologic cancer-related death in women around the world (1). The poor OV outcome is largely driven by the high rate of intraperitoneal dissemination and extraperitoneal metastasis in newly diagnosed cases (2). Survival rates for OV have only modestly changed for decades, and the 5-year survival rate remains below 45% (3). Therefore, it is very urgent to characterize the mechanisms and to identify the associated biomarkers for earlier diagnosis and treatment of OV.

More and more epidemiological evidences have suggested that a disturbed circadian rhythm was positively correlated with the risk of health disorders, such as obesity, cardiovascular disease, and cancer (4, 5). Biorhythms are essential for maintaining the whole-body homeostasis, while the disruption of biorhythms can cause the dysregulation of homeostasis and accelerate the development of diseases (6–8). Circadian rhythm disruption can contribute to suppress melatonin, which is a neurohormone known for its role in hindering cancer initiation and progression (9–11). Recent studies have demonstrated that circadian disruption might play a role in the etiology of cancers, including colorectal cancer, gastric cancer, prostate cancer, and breast cancer (12, 13). However, the mechanisms for the circadian rhythm in cancer are not well elucidated. One study has pointed out that circadian disruption led to circadian dysregulation of DNA repair genes and increased DNA damage and potentially elevated cancer risk (14).

*TIMELESS* (*TIM*), as a circadian clock gene, was originally identified in *Drosophila melanogaster* as an integral part of the circadian rhythm (15). TIM protein can interact with Cryptochrome (CRY) and Period (PER) proteins and has a negative effect on the circadian cycle (16, 17). In mammals, TIM is the best characterized gene in DNA replication and damage repair by controlling DNA replication and maintaining the stability of replication fork and genome (18, 19).

TIM has been reported to be highly expressed in various cancers and to be involved in the development and progression of cancer (20–22). TIM can regulate sphingolipid metabolism and promote tumor cell growth through Sp1/ACER2/S1P axis in breast cancer (23). TIM promotes the tumorigenesis of colorectal cancer by activating the β-catenin signal pathway and binding to Myosin-9, to induce its nuclear translocation (24). Accumulated studies have shown TIM has vital roles in modulating DNA damage, replication stress, and tumor growth (25). Enhanced levels of TIM and Claspin protect cancer cells from oncogene-induced replication stress in a checkpoint-independent manner, which is beneficial to tumor growth (26). However, the expression level and precise role of TIM and whether it can be treated as a novel biomarker for earlier diagnosis or treatment in OV are not defined yet.

In our study, we found that TIM expression was significantly increased in OV. Both *in vitro* and *in vivo* studies revealed that TIM promoted the OV cell growth. We have identified the genetic alteration of TIM in patients with OV. TIM co-expression network analysis revealed a wide effect of TIM on the immune cell infiltration and activation in OV. Furthermore, we showed the positive correlation between TIM expression level and the macrophages infiltration in OV. Thus, TIM might be a potential prognostic biomarker and immunotherapy target for OV patients.

## Materials and Methods

### Clinical Samples

Human ovarian cancer, ovarian cysts, and normal ovarian epithelium were obtained from either Department of Obstetrics and Gynecology, Fengxian Hospital, Southern Medical University, or Department of Gynecology, Changzhou Maternal and Child Care Hospital. None of them received radiotherapy, chemotherapy, and other related antitumor therapies before surgery. All human materials were obtained with informed content, and protocols were approved by the ethical review committee of the World Health Organization Collaborating Center for Research in Human Production (authorized by the Shanghai Municipal Government).

### Cell Culture and Reagents

Human ovarian cancer cell lines SKOV3, OVCAR3, ES-2, OV429, A2780, CAOV3, OVCAR8 were all preserved in Shanghai Cancer Institute, Ren Ji Hospital, School of Medicine, Shanghai Jiao Tong University. All cell lines were cultured in the indicated medium according to the ATCC protocols and supplemented with 10% (v/v) fetal bovine serum (FBS) and 1% antibiotics (100 μg/ml streptomycin and 100 units/ml penicillin) at 37°C in a humidified incubator with 5% CO_2_. All cell lines were tested negative for *Mycoplasma* contamination and authenticated with short tandem repeat assays.

### siRNA Transfection

Cells were plated at 60–70% confluence in 60 mm dishes. SKOV3 and OVCAR3 were transfected with si-TIM or with a non-targeted siRNA as a control. The siRNA oligonucleotides were synthesized from GenePharma (Shanghai, China), and the detailed sequences of the siRNA used in this study were shown as follows: si-TIM-1, 5’-GCUAGAGAUUGUCUCCCUUTT-3’;si-TIM-2, 5’-CCAAAUACAUCCUGGGCAATT-3’. Negative control was scrambled siRNA targeting no known gene sequence. The transfection steps were performed according to the manufacturer’s protocols using Lipofectamine^®^ RNAiMAX (Thermo Fisher Scientific, 13778030).

### Construction of Lentivirus Constructs

The recombinant lentivirus containing shRNA targeting TIM and vector controls were purchased from (GeneCopoeia). Cells were infected with 1 × 10^6^ recombinant lentivirus-transducing units in the presence of 6 μg/ml polybrene (Sigma, H9268). Twenty-four hours after infection, cells were selected and maintained with 2 μg/ml puromycin (Gibco, A1113802, USA). The knockdown efficiency of TIM was detected by qRT-PCR.

### RNA Isolation and qRT-PCR

Total RNA was extracted using Trizol reagent (Takara, a7603-1). PrimeScript RT-PCR kit (Takara, PR036A-1) was used to perform the RT according to the manufacturer’s protocol. Real-time qPCR was performed using Bestar^®^ SybrGreen qPCR master mix (DBI, DBI-2043) according to the manufacturer’s instructions. mRNA expression was normalized to endogenous GAPDH expression in the same sample. Quantitative real-time PCR was performed with an Applied Biosystems PRISM 7500 Sequence Detection System by using the default thermal cycling conditions: one initial cycle at 95°C for 10 min and 40 cycles of 15 s at 95°C plus 30 s at 60°C. Sequences of the primers were used to amplify genes as follows:

*TIM*-F: GTTTTGGCAATCTGCCTAAGGA,
*TIM*-R: GCAGCTCATACAAGGTTTCACT,
*GAPDH-F:* ACAACTTTGGTATCGTGGAAGG,
*GAPDH*-R: GCCATCACGCCACAGTTTC.

### Cell Viability Assay

SKOV3 and CAOV3 cells were plated in 96-well plates at a density of 3,000 cells per well with 100 μl of complete culture medium and cultured for 2–5 days. Each group contained four repeats. Ten μl Cell Counting Kit-8 (CCK-8, WST-8, Dojindo, Japan) solution was added to each well after 24, 48, 72, and 96 h and then cultured for an additional 1 h. The cell viability was measured at 450 nm with a Power Wave XS microplate reader (BioTek, Winooski, USA).

### Cell Migration

For the cell migration assay, 2.5 × 10^4^ cells were seeded into the upper chamber of the transwell plate (Millipore, USA). Cells were allowed to migrate for 24 h at 37°C. The migrated cells were then fixed and stained with 0.1% crystal violet, six randomly selected fields were photographed, and the cell numbers were counted.

### Immunohistochemistry Staining

IHC staining was performed as described previously (27). Briefly, the formalin-fixed and paraffin-embedded liver tissue slides were deparaffinized and rehydrated for histopathological evaluation. The sections were blocked in 10% BSA and then incubated with primary antibodies (TIM, Abcam ab72458; CD68, Servicebio, GB113150) overnight at 4°C and with secondary antibodies for 1 h at room temperature. Then the sections were treated with DAB substrate liquid (Thermo, S21024-2) and counterstained by hematoxylin. All the sections were observed and photographed with a microscope (Carl Zeiss).

### *In Vivo* Tumor Xenograft Model

Six-week-old female athymic nu/nu mice were randomly divided into two groups and injected subcutaneously in the lower back with a total of 2 × 10^6^ sh-NC or sh-TIM SKOV3/OVCAR3 cells in 150 μl PBS. Four weeks later, mice were sacrificed, and the tumor was isolated and the tumor weight was measured. All animals received humane care according to the local or national requirements for the care and use of laboratory animals.

### Data Mining Using TCGA, GTEx, TIMER, cBioportal

The gene expression data in OV were obtained using either GEPIA2 database (http://gepia2.cancer-pku.cn/#analysis) based on TCGA and GTEx cohort. The immunome of different immune cell types infiltrated into the tumors in OV was analyzed using Tumor Immune Estimation Resource (TIMER) 2.0 database (http://timer.cistrome.org/) (28). The LinkedOmics database (http://www.linkedomics.org/login.php) was applied to analyze the differentially expressed genes related to TIM from the TCGA-OV cohort through the LinkFinder module in the database (29). Function module analysis of Gene Ontology biological process (GO_BP), Kyoto Encyclopedia of Genes and Genomes (KEGG) pathways by the gene set enrichment analysis (GSEA) were performed in the LinkInterpreter module. The genetic alterations and the network module of TIM in OV were obtained from cBioPortal (www.cbioportal.org) (30). A protein-protein interaction network analysis was conducted to analyze the expression of TIM and the potential interactions through STRING (https://string-db.org/) (31). GeneMANIA (http://www.genemania.org) was also used to indicate the predicative values of TIM (32).

### Statistical Analysis

Data were presented as the means ± standard error of mean (SEM). Statistical analyses were done using GraphPad Prism 5 software (GraphPad Software Inc., San Diego, CA, USA). One-way ANOVA or two-tailed student’s T-test was used for comparison between groups. Values of P < 0.05 were considered statistically significant.

## Results

### TIM Expression Is Significantly Increased in Ovarian Cancer

**Figure 1:**
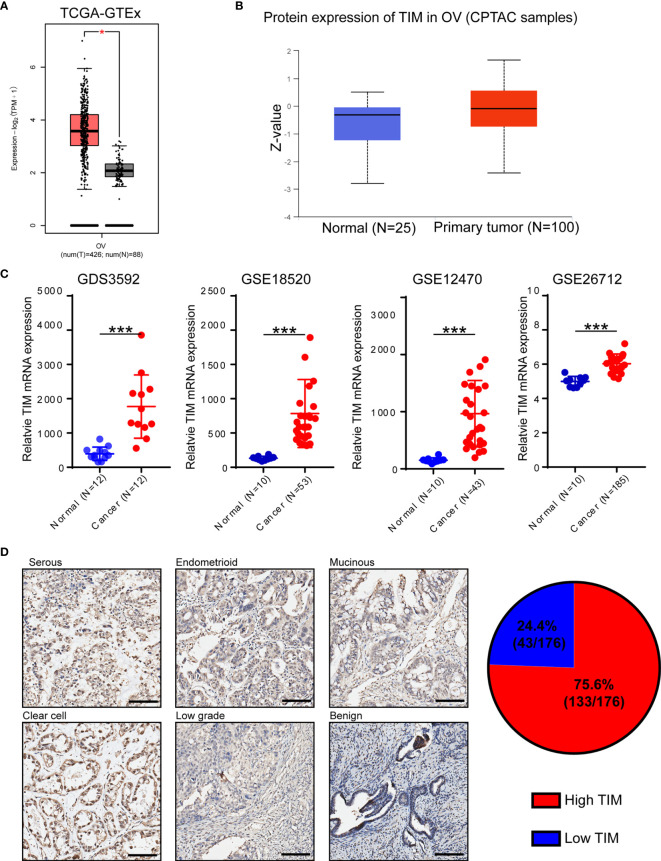
TIM expression is significantly upregulated in ovarian cancer (OV) patients. **(A)** Analysis of TIM mRNA expression in OV tissues compared to normal tissue using TCGA database. **(B)** Analysis of TIM protein expression in OV compared to normal tissue in CPTAC samples. **(C)** Relative mRNA level of TIM in OV patients using three independent gene expression omnibus (GEO) databases. **(D)** Left: Representative IHC staining showing the TIM expression in different types of ovarian cancer specimens as well as in benign ovarian cancer. Scale bar = 100 μm. Right: Graphical analyses showing the percentage of high/low expression of TIM level in a total of 176 OV patients. *p < 0.05, ***p < 0.001.

In order to identify which circadian clock-related gene plays vital roles during OV progression, we analyzed 16 circadian clock-related genes using TCGA database and found that TIM was the most significantly upregulated gene in OV (**Supplementary Figure 1**). Also, by using CCLE database, TIM mRNA was widely expressed in different tissues (**Supplementary Figure 2A**). To explore the distinct expression of TIM in different cancers, we first analyzed the level of TIM mRNA expression in pan-cancer *via* GTEx and TCGA and found increased TIM mRNA expression in the majority of cancers, indicating the important role of TIM during tumorigenesis and progression (**Supplementary Figure 2B**). TIM mRNA expression was significantly upregulated in ovarian cancer (OV) tissues compared to normal ovarian epithelial tissues (**Figure 1A**). Using CPTAC samples, increased TIM protein expression was also found in ovarian cancer tissues compared to normal tissues (**Figure 1B**). We also found the mRNA and protein expressions of TIM were related to the disease stages and tumor grades (**Supplementary Figures 2C–D**).

Furthermore, the gene expression data from four independent GEO databases (GDS3592, GSE18520, GSE12470, GSE26712) also showed that TIM mRNA expression was significantly increased in ovarian cancer samples (**Figure 1C**). We further verified the protein levels of TIM in 176 OV samples from several hospitals by immunohistochemical (IHC) analysis, and the results showed that the protein levels of TIM were upregulated in different types of ovarian cancer compared to benign tumors (**Figure 1D**). Among 176 ovarian cancer patients, 75.6% ovarian cancer tissues showed high TIM protein level, while 24.4% showed low TIM protein level (**Figure 1D**).

### TIM Knockdown Inhibited OV Cell Proliferation, Migration, and Invasion Both *In Vitro* and *In Vivo*

**Figure 2:**
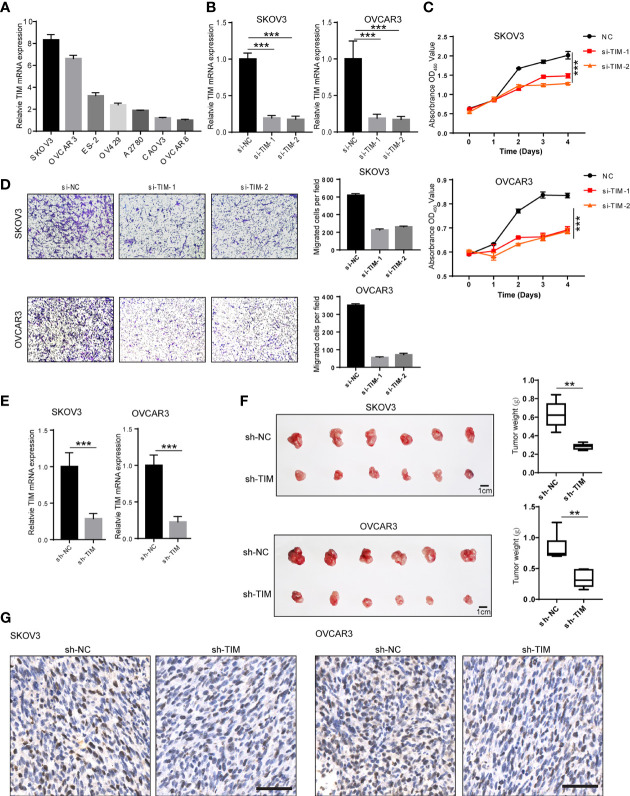
TIM promotes OV cell proliferation and migration both *in vitro* and *in vivo*. **(A)** The expression of TIM mRNA was detected by qRT-PCR in different OV cell lines. **(B)** The Interfere efficacy of TIM with either shRNA (si-TIM-1, si-TIM-2) or si-NC was analyzed by qRT-PCR. **(C)** The cell growth of SKOV3 and OVCAR3 in three groups (si-NC, si-TIM-1, si-TIM-2) was analyzed by CCK8 assay. **(D)** The migration of SKOV3 and OVCAR3 cells expressing si-TIM-1, si-TIM-2, or sh-NC was analyzed. **(E)** The interfere efficacy of TIM with either sh-TIM or sh-NC was analyzed by qRT-PCR in SKOV3 and OVCAR3 cells. **(F)** Subcutaneous xenografts transplanted with either SKOV3 or OVCAR3 cells expressing sh-NC and sh-TIM in nude mice (n = 6). **(G)** Ki67 staining of the subcutaneous xenograft tumors from both sh-TIM and sh-NC group. Scale bar =100 μm. **p < 0.01, ***p < 0.001.

To investigate the biological functions of TIM during OV progression, we first tested the mRNA of TIM in seven different OV cell lines including SKOV3, OVCAR3, ES-2, OV429, A2780, CAOV3, and OVCAR8 by qPCR. SKOV3 and OVCAR3 showed relatively high TIM expression, while CAVO3 and OVCAR8 showed relatively low TIM expression (**Figure 2A**). Therefore, we have chosen SKOV3 and OVCAR3, two cell lines, for subsequent functional experiments. To gain further insight into the oncogenic roles of TIM in OV, we knocked down the TIM expression by siRNA. We detected >80% decrease of TIM expression in mRNA levels by qRT-PCR (**Figures 2B, C**). CCK8 assay showed that cell proliferation of SKOV3 and OVCAR3 was significantly inhibited by TIM knockdown (**Figure 2C**). Also, cell migration was also inhibited by TIM knockdown (**Figure 2D**). Next, to explore the effect of TIM on the proliferation of OV cells *in vivo*, we stably knocked down TIM by short hairpin RNA (shRNA) in OV cells, which was verified in SKOV3 and OVCAR3 cell lines by qRT-PCR (**Figure 2E**). In the orthotopic xenograft mouse model, the tumor growth was obviously reduced in TIM knockdown group compared with control group, which was revealed by tumor weight (**Figure 2F**). IHC staining results showed that Ki67 staining was decreased in the tumors from TIM knockdown group compared to control group (**Figure 2G**). Taken together, those data suggested a promotive effect of TIM in OV growth both *in vitro* and *in vivo*.

### Genetic Alteration, Expression, and Interaction Analyses of TIM in Patients With OV

**Figure 3:**
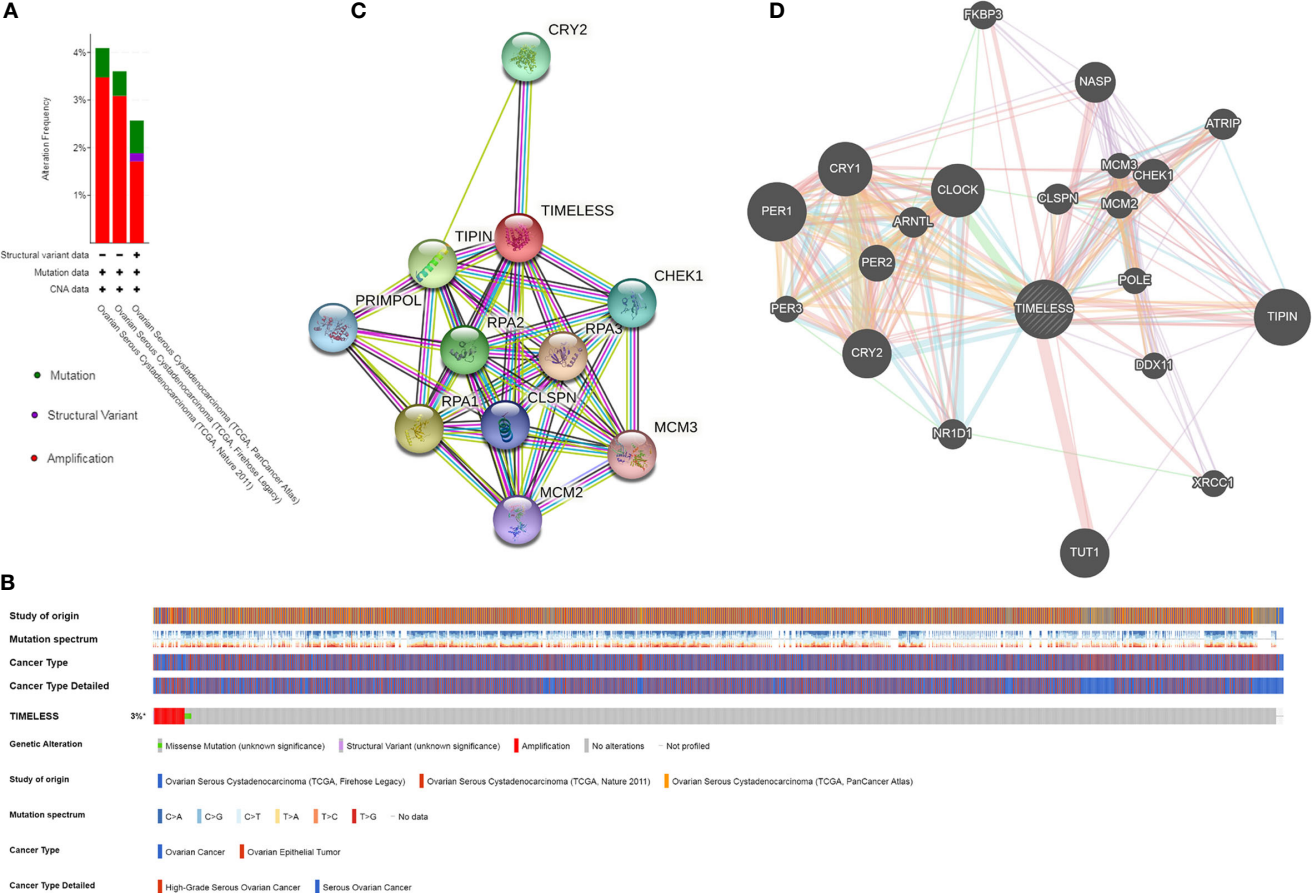
TIM gene mutation and expression analysis in OV. **(A, B)** The expression and mutation analysis of TIM in OV. TIM was altered in 51 samples (3%) of the total 1,680 patients with OV. **(C)** The protein-protein network of TIM was shown by STRING. **(D)** The protein interaction network for TIM using GeneMANIA. *p < 0.05.

The genetic alterations, correlations, and networks of TIM were analyzed using the cBioPortal online tool for OV. We found that two or more alterations were detected in TCGA-OV datasets (**Figure 3A**). TIM was altered in 51 samples (3%) of the total 1,680 OV patients (**Figure 3B**). Moreover, we conducted a protein-protein interaction analysis to explore the potential interactions with TIM with both STRING and GeneMANIA analysis (**Figures 3C, D**). The results showed the other circadian clock-related proteins, such as TIPIN, PER1, PER2, PER3, and CRY2, and DNA replication/repair-related proteins, such as CHEK1, RPA1, RPA2, and RPA3, were the potential interactive proteins with TIM. And those might be closely associated with TIM-related molecular functions.

### The Co-Expression Network of TIM in OV

**Figure 4:**
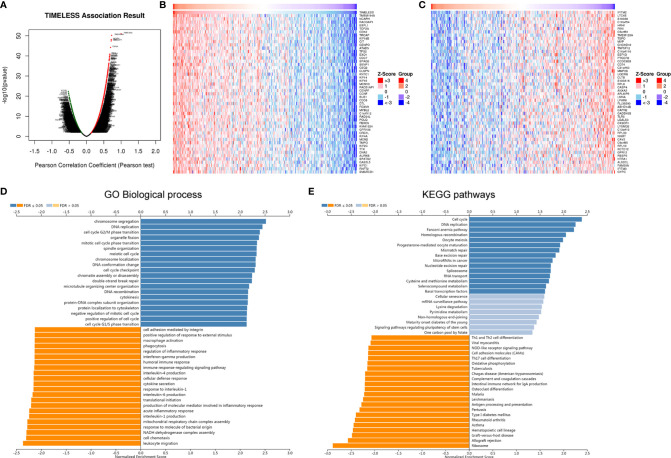
The co-expression genes with TIM in OV using online LinkedOmics database. **(A)** All significantly associated genes with TIM in TCGA-OV cohort were shown. Red represents positively linked genes, green represents negatively linked genes, and black indicates no correlated genes with TIMELESS. **(B)** Top 50 genes positively related to TIM in OV were shown in heatmap. Red represents positively linked genes, and blue represents negatively linked genes. **(C)** Top 50 genes negatively related to TIM in OV were shown in heatmap. **(D, E)** GO Biological process annotations and KEGG pathways of TIM in TCGA-OV cohort were shown.

For gaining more deep knowledge about the biological functions of TIM in OV, the LinkFinder module in the LinkedOmics database was applied to check the co-expression pattern of TIM in TCGA-OV cohort. We found that 6,313 genes (red dots) positively correlated with TIM, and 4,464 genes (green dots) negatively correlated with TIM as shown in **Figure 4A**. The heatmaps showed that the top 50 genes positively and negatively associated with TIM, respectively (**Figures 4B, C**). GO biological analysis indicated that the co-expressed genes of TIM mainly participated in cell cycle, cellular defense response, interleukin-4 production, interferon-gamma production, macrophage activation, leukocyte migration, etc. (**Figure 4D**). Furthermore, KEGG pathways analysis indicated the co-expressed genes of TIM were mainly enriched in cell cycle, cell adhesion molecules (CAMs), antigen processing and presentation, hematopoietic cell lineage, etc. (**Figure 4E**). Taken together, the above results show that TIMELESS co-expression network had a wide effect on the immune cell infiltration and activation in OV.

### TIM Expression Is Closely Correlated With Macrophages Infiltration in OV

**Figure 5:**
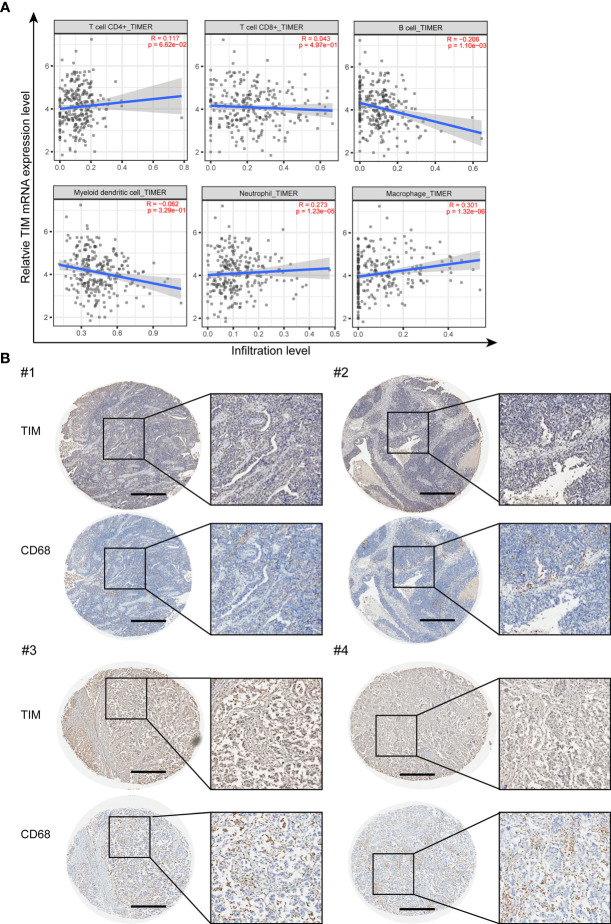
The infiltration of macrophages was closely associated with TIM expression in human samples. **(A)** The correlation between TIM expression levels and the infiltration of different immune cell subsets were analyzed in OV by using TIMER. **(B)** IHC staining of TIM and macrophage marker CD68 in OV tissue samples. Scale bar = 500 μm.

We further explored whether TIM can affect the immune cell infiltration in OV. The correlations between TIM expression level and different immune cell infiltration levels were analyzed using TIMER2.0 online database. The results showed that TIM has significant positive association with B cells, neutrophils, and macrophages, while no significant correlations with CD4^+^ T cells, CD8^+^ T cells, and dendritic cells (**Figure 5A**). As macrophages have the best correlation with TIM expression level in OV (**Supplementary Figure 3A**), therefore we focused on the macrophages and checked the macrophage infiltration by using the OV tissue microarray. IHC analysis of 176 tumor tissues from OV patients revealed a positive association between TIM expression and the number of CD68^+^ macrophages in OV tissues (**Figure 5B**). In addition, chemokines, including CCL2, CCL3, CCL4, CCL5, CCL18, and CCL20, play a vital role in macrophage recruitment. We identified that those chemokines were significantly upregulated in OV compared to normal tissue (**Supplementary Figure 3B**). Thus, TIM might exhibit a regulatory role on macrophages infiltration in OV.

## Discussion

Circadian rhythm disorders are positively correlated with the risk of cancer in many types of cancer (10, 33, 34). Recent studies have demonstrated that circadian clock genes are involved in tumorigenesis, metastasis, as well as in chemotherapeutic resistance (23, 24, 26, 35). Approximately 70% of OV are found to be involved in the metastases during initial diagnosis. Most patients die of malnutrition and intestinal obstruction secondary to the abdominal metastatic niche. TIM, a core gene related to circadian clock, is aberrantly upregulated in cancers, such as breast cancer and pancreatic cancer (36, 37).

TIM knockdown suppressed the self-renewal of cancer stem cells and the cancer cell invasion and migration abilities in breast cancer (38). Another study revealed that TIM regulated the sphingolipid metabolism through Sp1/ACER2/S1P axis, promoting the tumor cell growth of breast cancer (23). TIMELESS can inhibit the breast cancer cell invasion and metastasis by downregulating MMP9 expression (39). In colorectal cancer cells, TIM expression mediated by ERK suppressed G2/M arrest (20). Furthermore, TIM activation by H3K27 acetylation promoted the tumorigenesis of colorectal cancer by binding to Myosin-9 (24). Those provide us the evidences for the different ways of TIM in promoting tumorigenesis.

In this study, we identified a novel function of TIM in OV, which may provide a new basis for the treatment of OV. TIM is highly expressed in OV tissues compared with the corresponding normal tissues. Functional study showed that TIM knockdown inhibited the OV cell proliferation and migration. By using the cBioPortal online tool, the alterations were detected in OV. Also, we found the potential interacted proteins with TIM, which are the circadian clock-related proteins and DNA replication/repair-related proteins, suggesting TIM might play its role by interacting with those proteins. This requires further investigations. Co-expression network analysis suggested that TIM had a wide effect on the immune cell infiltration and activation in OV. Therefore, we focused on the infiltrated immune cells in the tumor microenvironment (TME) of OV for our following study.

TME plays a critical role in cancer cell proliferation, migration, and metastasis and could affect tumor progression and recurrence. TME is composed by cellular and non-cellular components, including fibroblasts, endothelial cells, different types of immune cells, together with cytokines, growth factors, and extracellular matrix (ECM) et al. (40–42). Immune cells in TME showed to harbor either tumor-promoting or tumor-suppressing activities. Our study found that TIM was positively associated with B cells, neutrophils, and macrophages, while no significant correlations with CD4^+^ T cells, CD8^+^ T cells, and dendritic cells, suggesting that TIM might also affect the immune status. Therefore, it is worth to further investigate the role of TIM in the field of tumor immunotherapy.

The most frequently found immune cells within the TME are tumor-associated macrophages (TAMs), which are necessary to drive tumor progression, invasion, and metastasis (43–45). We found that macrophages have the best correlation with TIM expression in OV. By IHC staining of OV microarray, we verified the positive correlation between TIM expression and the macrophages infiltration in OV. However, how TIMELESS influences the macrophage infiltration during OV progression and the underlying mechanism are still under investigation. Previous studies have shown that TIM is involved in regulating the phagocytosis of bacteria in *Drosophila* (46). Whether the phagocytosis of macrophages is regulated by TIM needs to be further investigated, which will provide us more information for the macrophage function affected by TIM. In addition to the phagocytosis of macrophages, TIM might have impact on the macrophage migration and invasion, which also need to be further investigated.

In conclusion, we performed the comprehensive analysis of the expression and immune cell infiltration for TIM in ovarian cancer and revealed that TIM had an effect on the immune cell infiltration during OV, especially for macrophages. Thus, TIM might be used as a potential prognostic biomarker, and targeting TIM might constitute a new approach for the immunotherapy intervention of OV.

## Data Availability Statement

The raw data supporting the conclusions of this article will be made available by the authors, without undue reservation.

## Ethics Statement

The studies involving human participants were reviewed and approved by the World Health Organization Collaborating Center for Research in Human Production (authorized by the Shanghai Municipal Government). The patients/participants provided their written informed consent to participate in this study. The animal study was reviewed and approved by the East China Normal University Animal Care Commission. Written informed consent was obtained from the owners for the participation of their animals in this study.

## Author Contributions

RZ conceived the project, and XX, DL and ZW designed the experiments and interpreted data in the manuscript. XX, FG, LH and XC performed the experiments. XX and DL performed bioinformatics analyses and wrote the manuscript. DL, ZW and RZ edited the manuscript. All authors contributed to the article and approved the submitted version.

## Funding

This study was supported by grants from Fengxian District Science and Technology Commission Project (No. 20181802 to XX), Shanghai Municipal Health Commission (No. 201940506 to XX), and National Natural Science Foundation of China (No. 81974407 to RZ).

## Conflict of Interest

The authors declare that the research was conducted in the absence of any commercial or financial relationships that could be construed as a potential conflict of interest.

## Publisher’s Note

All claims expressed in this article are solely those of the authors and do not necessarily represent those of their affiliated organizations, or those of the publisher, the editors and the reviewers. Any product that may be evaluated in this article, or claim that may be made by its manufacturer, is not guaranteed or endorsed by the publisher.
