# Supplementary material for: TIMELESS Promotes Tumor Progression by Enhancing Macrophages Recruitment in Ovarian Cancer
Source: Front Oncol. 2021 Aug 19;11:732058. doi: 10.3389/fonc.2021.732058 (PMC8417241; doi:10.3389/fonc.2021.732058)
Supplement: Supplementary file 1 [file DataSheet_1.pdf]

**Supplementary Figures and Figure legends**

Sup Figure.1

| Genes | logFC(T/N) | p value |
|---|---|---|
| *CLOCK* | 0.108357178 | 0.039 |
| *NPAS2* | 0.471967788 | 3.96E-04 |
| *ARNTL* | -0.015782997 | 0.532 |
| *PER1* | -0.773996325 | 0.988 |
| *PER2* | -1.230817842 | 1 |
| *PER3* | 0.182692298 | 0.217 |
| *CRY1* | -0.126972856 | 0.8 |
| *CRY2* | -1.27857955 | 1 |
| ***TIMELESS*** | **1.779890039** | **2.30E-09** |
| *CSNK1D* | -0.837539634 | 1 |
| *CSNK1E* | 0.200378798 | 0.045 |
| *RORA* | -0.249748715 | 0.938 |
| *RORB* | 0.077242999 | 0.023 |
| *RORC* | -0.128293401 | 0.909 |
| *NR1D1* | -0.069014678 | 0.114 |
| *NR1D2* | 0.352758525 | 0.18 |

**Sup Figure.1** . The analysis of 16 circadian clock-related genes in OV using TCGA datasets. T: Tumor; N:normal.

Sup Figure.2

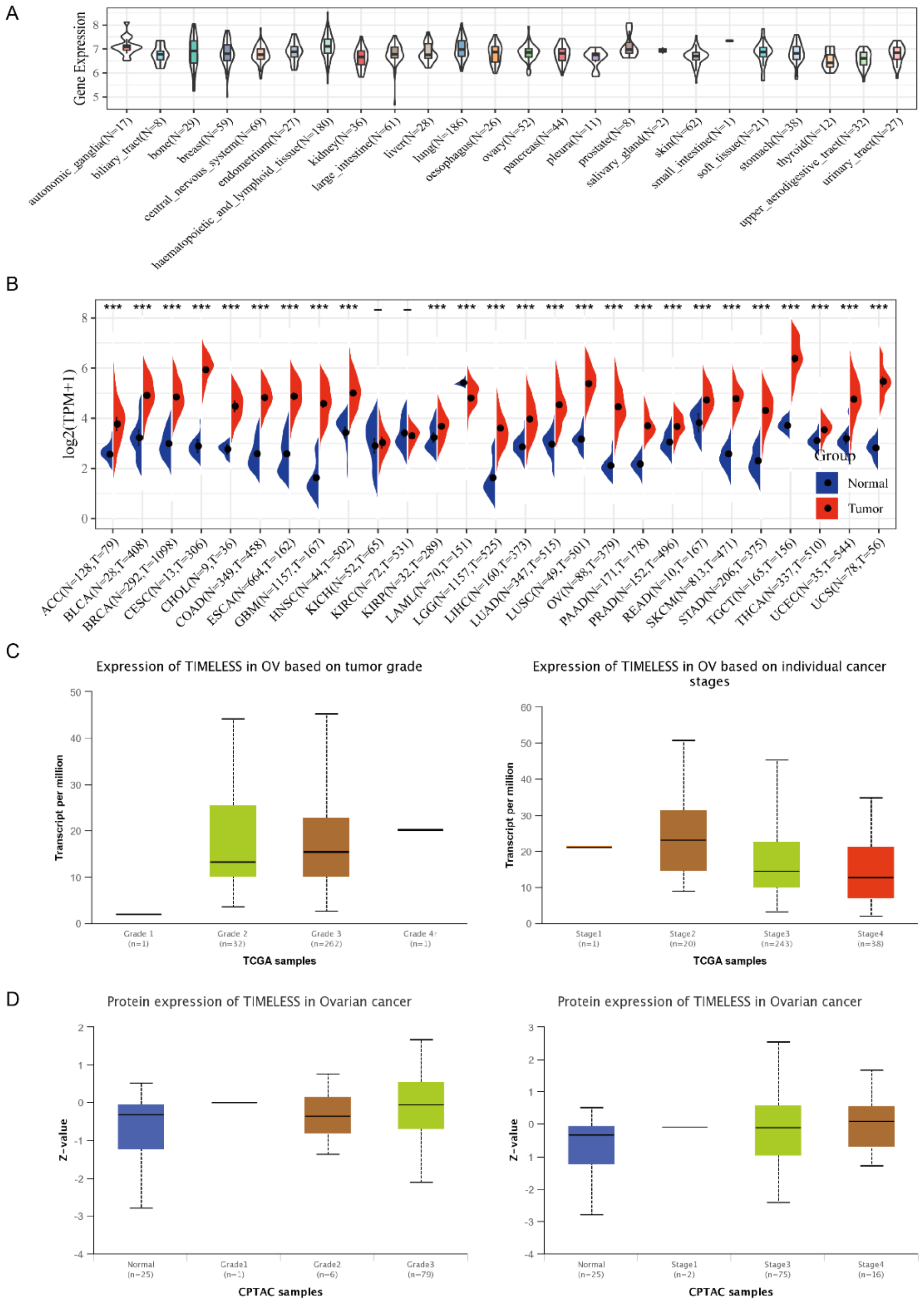


**Sup Figure. 2**. The analysis of TIM mRNA and protein expression. (A) Analysis of TIM mRNA expression in different tissues. (B) Comparison TIM mRNA expression in tumor tissues compared to normal tissues in Pan-cancer. (C) Analysis of TIM mRNA expression in different tumor grades and disease stages of OV using CPTAC samples. (D) Analysis of TIM protein expression in different tumor grades and disease stages of OV using CPTAC samples.

Sup Figure.3

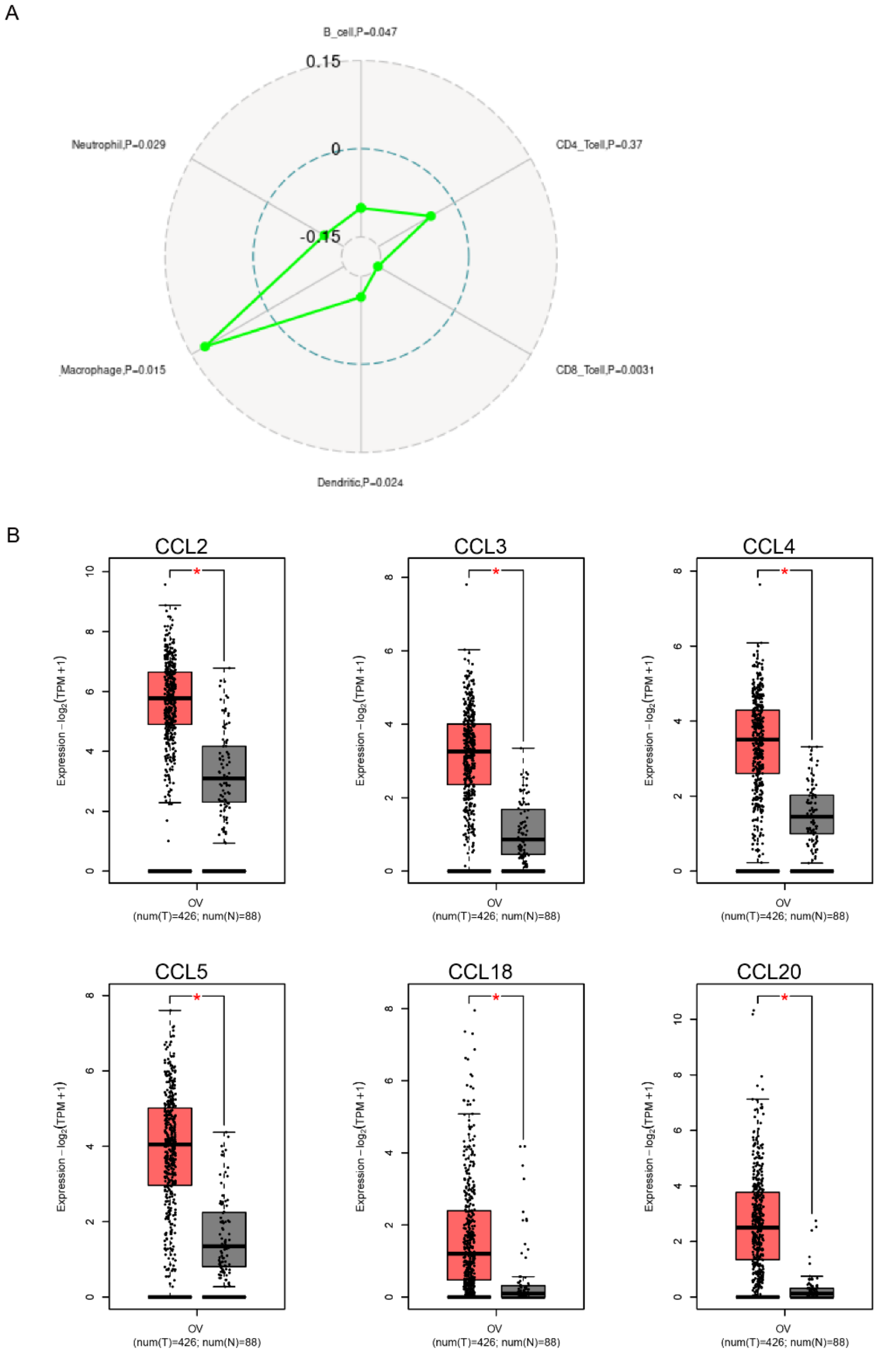


**Sup Figure. 3.** The analysis of tumor immune microenvironment in OV. (A) The correlation analysis between TIM expression and different immune cell infiltration. (B) The mRNA expression levels of several chemokines in OV and normal tissue were analyzed by TIMER.
